# Does polyandry control population sex ratio via regulation of a selfish gene?

**DOI:** 10.1098/rspb.2013.3259

**Published:** 2014-05-22

**Authors:** Tom A. R. Price, Amanda Bretman, Ana C. Gradilla, Julia Reger, Michelle L. Taylor, Paulina Giraldo-Perez, Amy Campbell, Gregory D. D. Hurst, Nina Wedell

**Affiliations:** 1 Biosciences, College of Life and Environmental Sciences, University of Exeter, Cornwall Campus, Penryn TR10 9EZ, UK; 2 School of Biology, University of Leeds, Leeds LS2 9JT, UK; 3 Centro de Biología Molecular ‘Severo Ochoa’ (CSIC-UAM), Universidad Autónoma de Madrid, Cantoblanco, 28049 Madrid, Spain; 4 Department of Animal and Plant Sciences, University of Sheffield, Sheffield S10 2TN, UK; 5 Institute of Integrative Biology, University of Liverpool, Liverpool L69 7ZB, UK

**Keywords:** polyandry, sexual selection, sex ratio distorter, sperm competition, meiotic drive, geographical cline

## Abstract

The extent of female multiple mating (polyandry) can strongly impact on the intensity of sexual selection, sexual conflict, and the evolution of cooperation and sociality. More subtly, polyandry may protect populations against intragenomic conflicts that result from the invasion of deleterious selfish genetic elements (SGEs). SGEs commonly impair sperm production, and so are likely to be unsuccessful in sperm competition, potentially reducing their transmission in polyandrous populations. Here, we test this prediction in nature. We demonstrate a heritable latitudinal cline in the degree of polyandry in the fruitfly *Drosophila pseudoobscura* across the USA, with northern population females remating more frequently in both the field and the laboratory. High remating was associated with low frequency of a sex-ratio-distorting meiotic driver in natural populations. In the laboratory, polyandry directly controls the frequency of the driver by undermining its transmission. Hence we suggest that the cline in polyandry represents an important contributor to the cline in *sex ratio* in nature. Furthermore, as the meiotic driver causes sex ratio bias, variation in polyandry may ultimately determine population sex ratio across the USA, a dramatic impact of female mating decisions. As SGEs are ubiquitous it is likely that the reduction of intragenomic conflict by polyandry is widespread.

## 1. Introduction

Variation in female mating frequency in nature is profound. Females of some species mate only once in their life, whereas others may mate with hundreds of males [1]. Research in the last 30 years suggests that the frequency at which females remate is of key importance in the ecology and evolution of many animals [2]. The frequency of polyandry can affect the level of gene flow and the effective population size [3], the population viability [4,5], and the intensity of post-copulatory sexual selection and sexual conflict [6], giving rise to a range of adaptations and counter-adaptations for the manipulation of mates and rivals [2,7]. In addition, polyandry can reduce within-family relatedness and thereby inhibit within-family cooperation, thus affecting the intensity of parent–offspring conflict [8], and the evolution of cooperation and sociality [9,10]. However, theory and experimental evolution studies also indicate that polyandry can promote harmony within the genome, through undermining the spread of selfish genetic elements (SGEs) [11,12].

SGEs are genes that subvert the normal patterns of inheritance to increase their representation in subsequent generations [13,14]. SGEs are ubiquitous in living organisms [13] and can make up a large proportion of the genome, and the intragenomic conflicts they create are thought to have had major impacts on the evolution of sex and reproductive systems [13,15]. Recent discoveries of novel SGEs in well-studied species [16–18] suggest that a vast array of SGEs remain to be discovered. Models of the dynamics of many SGEs suggest that they should spread rapidly through populations, but most empirical studies have found their abundance to be stable in nature [13,14]. Although many mechanisms have been suggested to control the frequency of SGEs, in most cases we do not understand how their abundance is determined in natural populations [14,19]. Many SGEs, including meiotic drive elements, B chromosomes, endosymbionts and some transposons, often target male gametes and have been shown to impair male fertility through manipulation of spermatogenesis [20]. This reduces the success of males carrying the SGE during sperm competition [20]. Polyandry is therefore likely to reduce the transmission of any SGEs that reduce the sperm competitive ability of males [11]. Hence it is possible that differences in degree of polyandry are important in determining the frequency of many SGEs in the wild. However, this hypothesis has never been tested, because it requires intra-specific variation in the level of polyandry.

*Sex ratio* (*SR*), a meiotic driving X chromosome, has been studied in the fruitfly *Drosophila pseudoobscura* for more than 75 years [21]. The biology of this SGE indicates that its population frequency should be vulnerable to control by polyandry. *SR* has little consistent effect in females [22], but in males, *SR* causes the death of all Y-chromosome-bearing sperm during spermatogenesis [23], leaving all functional sperm carrying only the *SR* X chromosome. Hence all offspring of *SR* males are daughters, and inherit the *SR* X chromosome. As there is no genetic resistance to sex ratio drive in *D. pseudoobscura* [24], simple models predict that *SR* should spread rapidly through populations until it causes extinction owing to a lack of males [21,25]. In nature, the abundance of *SR* in *D. pseudoobscura* is broadly stable, having been historically common in populations in the southern USA, reaching frequencies of 30% at the US–Mexican border, and becoming rarer to the north, being absent in Canada [21,26]. This latitudinal cline in *SR* frequency has never been explained [27]. However, the loss of sperm by *SR*-carrying males makes them poor sperm competitors [28], raising the possibility that polyandry could regulate the frequency of *SR* by undermining its transmission, as predicted by Haig & Bergstrom [11]. Theory predicts that the transmission advantage of *SR* should be highest in monandrous populations, and that a sufficiently high frequency of sperm competition could prevent the spread of *SR* or eliminate it [29]. Experimental work has confirmed that allowing females just one additional mating is sufficient to prevent the spread of *SR* through laboratory populations, and that *SR* spreads rapidly and causes population extinction when female remating is prevented in as little as nine generations [4]. So polyandry can directly regulate *SR* in experimental populations, making polyandry a strong candidate for influencing the distribution of *SR* in nature.

To investigate the hypothesized link between polyandry and meiotic drive frequency in the wild, we determined the frequency of multiple paternity in seven natural populations of *D. pseudoobscura*. We then estimated the frequency of *SR* in these populations and tested for the predicted negative association between *SR* frequency and the rate of polyandry.

## 2. Material and methods

### (a) Estimate of polyandry in wild females

We caught flies using standard banana baits [30] from seven locations across the USA in May–June 2008 (table 1; electronic supplementary material, figure S1). The seven locations were suitable forest habitat separated by areas of unsuitable habitat, such as desert or pasture. Most collections were carried out in National Forests, and no permits or licences were required. Two collection sites were on private land, and permission was given by the landowners for this. Baits were placed under trees, and emptied at dawn and dusk to reduce the likelihood that high densities at the bait would influence female mating frequency. Females were caught, isolated from males and sent to the laboratory, where they were maintained at 23°C, with a 14 L : 10 D cycle on standard *Drosophila* food [31]. We genotyped each wild-caught female and up to 22 of her randomly chosen offspring (range 9–22 offspring, median of 21 offspring per family, 189 families) using four highly polymorphic microsatellites (methods detailed in [32], microsatellites described in [33]). The number of sires was assayed by subtracting the maternal genotype from that of each offspring, and, for the most variable locus, dividing the number of remaining alleles by 2 to give a minimum number of fathers [34].

**Table 1.** The locations of the seven populations and the percentage of wild-caught females found to have mated with more than one male.

population	location	state	latitude north	longitude west	% multiple paternity
1	Chiricahua mountains	Arizona	31°54′55″	109°15′95″	58
2	Show Low	Arizona	34°07′37″	110°07′37″	52
3	Mount Lemmon	Arizona	32°21′95″	110°41′66″	73
4	Zion Forest	Utah	37°25′91″	113°03′13″	59
5	Panguitch	Utah	37°55′87″	112°19′66″	73
6	Fillmore	Utah	38°55′86″	112°14′60″	73
7	Lewistown	Montana	47°04′47″	109°16′53″	92

One potential problem in assessing multiple paternity across populations is that allele frequencies typically differ between populations, meaning that the power of each locus to detect multiple paternity also differs between populations. If one population has few alleles, or one very common allele, then many males will share this allele, and detecting multiple paternity will be difficult, creating an artificially low rate of detected multiple paternity. To assess whether this could bias our results, we calculated the chance of failing to detect multiple paternity in each family, following Harshman & Clark [35]. This method calculates the probability of misidentifying multiple paternity as single paternity by combining the probabilities of the two males being identical in genotype, the two males sharing one common allele that is not represented in the offspring sampled, and the two males having no common alleles but only half the alleles being represented in the sample offspring. Using the population allele frequencies for each population derived from the maternal genotypes and samples of either nine offspring (the minimum sampled per real family) or 22 offspring (the majority of families sampled), the probability of misidentifying a multiply sired family was less than 0.00002 for every population. We also used the Gerudsim application of Gerud [36] to simulate families consisting of a mother, two fathers and nine offspring (three descended from one father, six from the other) using the allele frequencies found in each population. For each population, we ran 10 separate parental simulations with 1000 iterations (i.e. simulations of offspring genotypes). The mean probability of failing to detect multiple paternity owing to similar genotypes in two males was 0.4% per family, with the least successful population having a failure rate of 1.08% per family. Considering that this estimate is derived from the highly conservative assumption of only nine offspring per family (the fewest we were able to genotype for one of the families), rather than the 21 offspring used for most families, it is unlikely that these errors play a significant role affecting our results. Furthermore, the three southernmost populations, where we found the lowest rates of multiple paternity, had lower likelihoods of failing to detect multiple paternity than the three northernmost populations, so errors of this kind would mask the latitudinal cline we detected, rather than create it.

### (b) Remating propensity of granddaughters in the laboratory

We collected one virgin daughter of 8–15 wild-caught females (see above) from each population (total 71 wild-caught females) and placed them in a standard *Drosophila* vial with a male sibling. Each pair was moved on to a new vial twice each week. We collected 8–10 virgin female offspring from each pair (the F_2_ granddaughters of the wild-caught females). At 3 days old, we placed each F_2_ granddaughter in an individual *Drosophila* vial, and left her overnight to acclimatize. The following day, we presented each F_2_ granddaughter with a single stock male for 2 h. These males were 3-day-old virgins collected from a stock mass population, all carrying the non-driving X chromosome *standard* (*ST*), derived from a collection carried out at Show Low, Arizona, USA, in 2004. We watched the vials continuously, and noted all copulations. Females that failed to mate were excluded from the experiment (264 of 748 females). Failure to mate with the first male did not correlate with the latitude of the population of origin (Spearman's correlation: *n* = 7 populations, coefficient = 0.286, *p* = 0.535), nor with the remating propensity of siblings (Spearman's correlation: *n* = 7 populations, coefficient = 0.071, *p* = 0.879). Hence we concluded that failure to mate with the initial male was stochastic, and could be disregarded in the rest of the analysis, as is standard in *Drosophila* remating trials. After a successful first copulation, we removed the male. Each day for the following 6 days, we presented the females with a new virgin stock male carrying *ST*. All vials were watched as before, and all rematings were noted. We blinded the experiment by labelling each female with a randomly assigned number, and the observers were not aware of the genotype of the individuals. All flies were moved by aspiration to avoid anaesthesia [37]. Remating was scored in two ways: first, whether or not the female remated at all over the 6 days; second, analysing only those females that remated, we examined mean number of days to remating. To test whether the results of the above experiments could have been due to the influence of maternal effects or differential response to the tester strain, we maintained isolines from two populations (Show Low, the southern population; and Lewistown, the northernmost population), and tested for remating propensity after 40 generations in the laboratory. At least 30 females from each of 23 isolines were tested as above, but were mated to males from their own population, and the mating assays were conducted simultaneously.

### (c) Sperm competitive ability in northern and southern populations

Our previous estimate of the relative sperm competitive ability of *SR* and *ST* males was carried out using flies from a single southern population, collected at Show Low [28]. If *ST* males have lower success in sperm competition when competing against *SR* males, this would make polyandry less effective at reducing the transmission of *SR* in northern populations. However, if northern *ST* males are more successful in sperm competition against *SR* males than southern *ST* males, then this would increase the likelihood that polyandry prevents *SR* colonizing northern populations. To examine this, we established mass populations of *SR* and *ST* flies from the Show Low population by crossing individuals from 20 isolines from this population, and a mass Lewistown *ST* population by crossing 16 isolines from Lewistown. After two generations of free mating in each population, we collected homozygous *ST*/*ST* females from the Show Low population and mated them to both an *SR* male and an *ST* male, using the methodology described by Price *et al.* [28]. In brief, we mated 4-day-old virgin females to a male, and 3 days later she was mated to a second male, and then allowed to oviposit for 6 days. In half the trials, the *ST* male was from the Show Low population, whereas the *SR* males were always taken from the Show Low population, to represent the situation that occurs when *SR* and *ST* males compete in Show Low, and to represent an *SR* male immigrating into a northern population. The order of mating was randomized. Females that did not mate twice were discarded. After the second mating, females were allowed to oviposit for 12 days, and the resulting offspring were collected. The offspring were counted by sex, as all sons were fathered by the *ST* male, and 23 of the daughters were genotyped for *SR* [32]. We measured the success of the *ST* males in sperm competition by calculating the number of offspring that inherited *SR* by multiplying the number of daughters produced by the proportion of the 23 daughters that carried *SR*. However, as a conservative measure we also independently analysed the proportion of both sons and daughters inheriting *SR*.

### (d) Correlation of polyandry and sex ratio frequency in nature

The distribution of *SR* across the USA was ascertained from collections between 1938 and 1957 by Dobzhansky [26], and our own collections in 2004 and 2008. We assayed *SR* frequency in our populations by genotyping at least 50 wild-caught males and females from each population. We detected *SR* and *ST* chromosomes using markers described by Price *et al*. [32]. The data from our collections in 2004 for Show Low and Lewistown were pooled with the data from 2008 for these locations. In 2004, we also carried out a collection at Flagstaff (35°05′00″ N, 111°44′10″ W), which was not repeated in 2008. This was used as a single data point. The data were analysed using a generalized linear model, with latitude as a fixed effect and survey as a random effect, using a normal error distribution and an identity link function. We used stepwise removal of factors from the full model to produce a final minimal model [38]. Analysis was conducted using R v. 2.13.1 [39].

## 3. Results

### (a) Polyandry in wild females from populations across the USA

**Figure 1.**
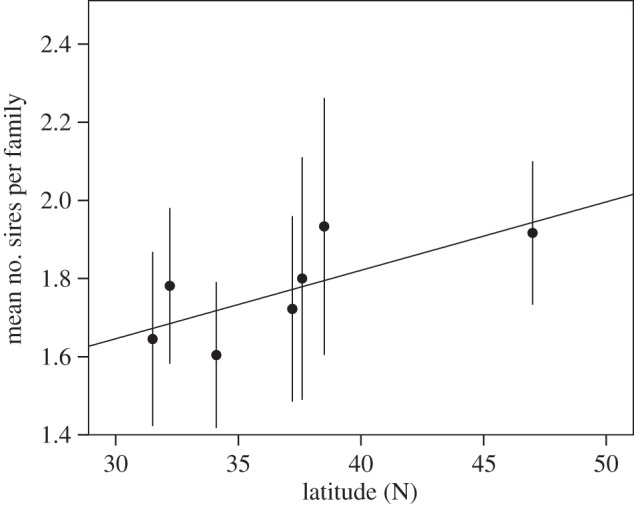
The mean number of sires detected in the broods of wild-caught females, with 95% confidence intervals. More sires are detected at higher latitudes (Spearman's correlation: all females: 12–40 broods from each of seven populations; *n* = 7, coefficient = 0.786, *p* = 0.036; analysing non-SR females only does not change the rank order: 12–34 broods from each of seven populations; *n* = 7, coefficient = 0.786, *p* = 0.036).

We first surveyed the frequency of multiple paternity in seven populations across the USA, ranging from southern Arizona to Montana (table 1). We observed that the mean number of sires per family was significantly correlated with latitude, with southern populations having fewer sires per brood than northern populations (figure 1). Carrying the *SR* chromosome does not affect the remating propensity of female carriers [27], and indeed the significance of the polyandry cline described above was not changed after re-analysis using only the non-*SR* females (figure 1).

### (b) Remating propensity of granddaughters in the laboratory

**Figure 2.**
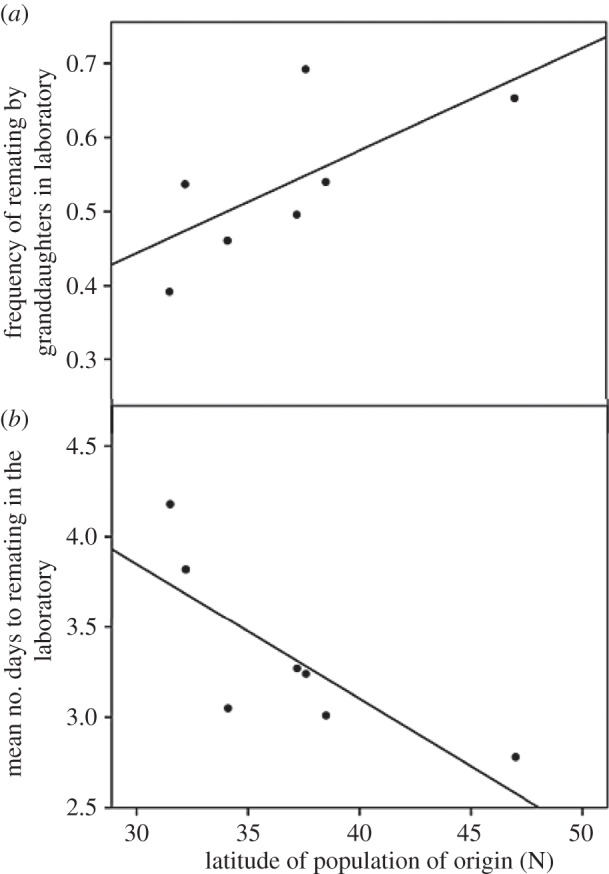
The remating propensity of females derived from seven populations across the USA. (*a*) The frequency of remating in the laboratory by the F_2_ granddaughters of each wild-caught female is positively correlated with the latitude of the population from which they were descended (10–12 families from each of seven populations, 484 females in total; Spearman's correlation: *n* = 7, coefficient = 0.786, *p* = 0.036). (*b*) The mean number of days to remate of the F_2_ granddaughters of each wild-caught female is negatively correlated with the latitude of the population from which they were descended (mean of 10–12 families from each of seven populations, 361 females total; Spearman's correlation: *n* = 7, coefficient = −0.893, *p* = 0.007).

To investigate the genetic component of the cline in polyandry observed, we examined the remating propensity of the F_2_ granddaughters of the wild-caught females. The granddaughters of females from southern populations were significantly less likely to remate than the granddaughters of females from northern populations (figure 2*a*). Where remating occurred, the granddaughters of southern females also showed a significantly longer delay to remating than the granddaughters of northern females (figure 2*b*). This pattern was replicated 40 generations after collection, with females from a southern population having significantly lower remating propensities than females from the northernmost population (*t* test: *n* = 23, *t* = 2.326, *p* = 0.032), indicating the difference observed in the granddaughters was not because of maternal effects.

### (c) Sperm competitive ability in northern and southern populations

*SR* males were significantly worse sperm competitors than *ST* males from both populations, irrespective of mating order (proportion of offspring fathered by the *SR* male when competing with northern *ST* male: *SR* mated first: 0.06, *n* = 27; *SR* mated second: 0.31, *n* = 22; when competing with a southern *ST* male: *SR* mated first: 0.23, *n* = 20; *SR* mated second: 0.53, *n* = 26). *SR* males were significantly worse as sperm competition when mating in the first male role (*F*-test: *F*_1,92_ = 25.148, *p* < 0.001). *ST* males from the northern Lewistown population were significantly more successful in sperm competition than *ST* males from Show Low (*F*-test: *F*_1,92_ = 12.398, *p* < 0.001). However, there was no population difference in the sperm competitive success of non-*SR* males in relation to mating order (no interaction between non-*SR* male population of origin and mating order: *F*-test: *F*_1,92_ = 0.192, *p* = 0.662). The results were qualitatively the same when proportion of sons or proportion of daughters fathered by the non-*SR* male were analysed. The proportion of sons and daughters that were found to carry *SR* were significantly negatively correlated (Spearman's rank correlation of proportion of sons and proportion of daughters that inherited *SR*: *n* = 95, *ρ* = −0.619, *p* < 0.001), indicating that both measures were accurate, and that the combined estimate of the proportion of offspring inheriting *SR* was correct.

### (d) Correlation of polyandry and frequency of sex ratio

**Figure 3.**
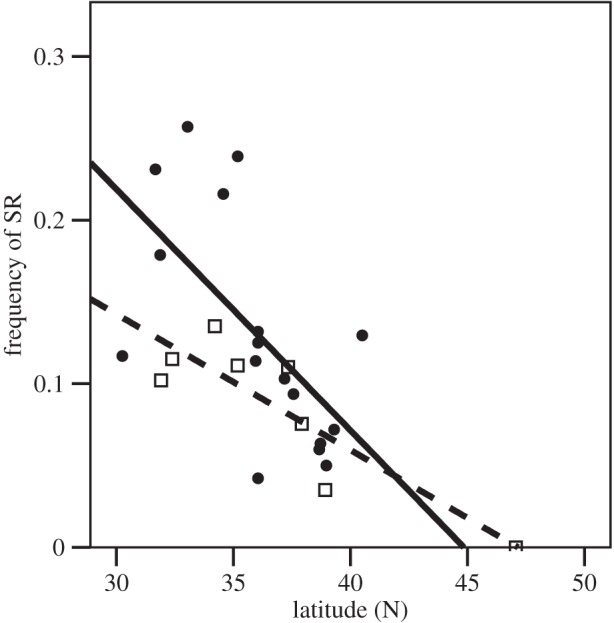
The frequency of *SR* and latitude across the USA. *SR* is more abundant in the southern USA, in surveys conducted by Dobzhansky [26] (solid circles, solid fit line) and in our eight populations (collected 2004–2008; hollow squares, dashed fit line).

We estimated the frequency of *SR* in our collections and tested whether *SR* frequency was correlated with latitude, both in our surveys and historical data from 1940 to 1958 [26]. The frequency of *SR* was negatively correlated with latitude across populations when both historical and contemporary data were pooled (figure 3; *F*-test: *F*_1,23_ = 16.284, *p* < 0.001), and in each dataset when analysed separately (*F*-tests: Dobzhansky data: *F*_1,15_ = 9.571, *p* = 0.007; our data: *F*_1,7_ = 19.474, *p* = 0.005). Furthermore, there was no evidence that the relationship between the frequency of *SR* and latitude had changed over the 70 years between the surveys (*F*-tests: effect of survey date—post-2004 versus pre-1958—on the relationship between latitude and SR frequency: *F*_1,22_ = 1.287, *p* = 0.269; effect of survey date *per se*: *F*_1,21_ = 2.728, *p* = 0.113), confirming that this latitudinal cline in *SR* frequency has remained broadly stable for at least 70 years.

**Figure 4.**
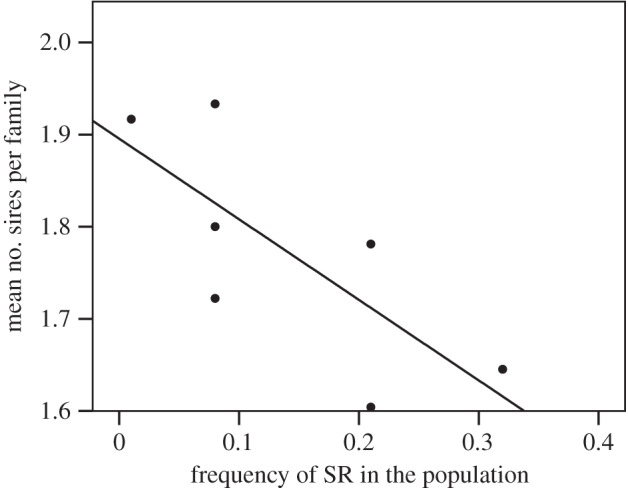
The number of fathers detected in the broods of wild-caught females and *SR* frequency. Mean number of sires is negatively correlated with the frequency of *SR* across natural populations (mean of 12–40 families from each of seven populations, total 189 families; Spearman's correlation: *n* = 7, coefficient = −0.821, *p* = 0.023).

The mean number of sires within the broods of field-caught females was negatively correlated with *SR* frequency across populations (figure 4; Spearman's correlation: *n* = 7, coefficient = −0.821, *p* = 0.023). Overall, the median number of sires per mother ranged from 1.62 (southern population, *SR* at 30% frequency) to 1.96 (northern population, *SR* near absent), a close match to the estimate that two mating opportunities is enough to cause the frequency of *SR* to decline in laboratory populations [4]. Moreover, the median number of fathers per brood was significantly lower than two in only the four southernmost populations, where *SR* was most common (one sample one-tailed Wilcoxon signed-ranks test: Chiricahua: *n* = 31, *W* = 16, *p* = 0.003; Show Low: *n* = 40, *W* = 56, *p* < 0.001; Mt Lemmon: *n* = 32, *W* = 12, *p* = 0.018; Zion Forest: *n* = 36, *W* = 52.5, *p* = 0.013; Panguitch: *n* = 19, *W* = 4, *p* = 0.09; Fillmore: *n* = 19, *W* = 16, *p* = 0.353; Lewistown: *n* = 12, *W* = 0, *p* = 0.159). Taken together, our results strongly support the hypothesis that variation in polyandry controls the frequency of *SR* in nature, as previously demonstrated in the laboratory [4].

## 4. Discussion

These results demonstrate first that there is a latitudinal cline in female remating frequency in *D. pseudoobscura*, and second that there is a genetic component to this cline. To the best of our knowledge, this is the first heritable geographical cline in female remating frequency to be discovered. The relatively high levels of gene flow between populations of *D. pseudoobscura* across the USA [40] make it unlikely that the genetic component of this cline is owing to historical factors, but rather is probably maintained by current selection. The cline is likely to be a result of selection on female mating behaviour, as the differences were expressed when females were reared under common garden conditions and mated with standard stock males. At present, the cause of this cline in polyandry in nature is not known.

Both methods of estimating population-level polyandry can be criticized. For example, the wild-caught females were of unknown age, and if females in the north live longer, this could result in higher numbers of brood sires. In addition, there are many well-known potential sources of error in genotyping the offspring of wild-caught females. Similarly, if northern females are adapted to cooler temperatures than southern females, then exposure to the standard 23°C laboratory temperature might lead to differences in female mating behaviour in the laboratory. However, using two independent methods to determine degree of polyandry makes it unlikely that our results are artefactual, which is further corroborated by the significant correlation of laboratory and field measures across populations (Spearman's correlation: *n* = 7 populations, *r*_s_ = 0.821, *p* = 0.023). One additional risk is that an inability to detect *SR* sires owing to their poor sperm competitive ability might cause us to miss *SR* sires in some populations, hence generating the observed polyandry cline. However, *SR* is typically found at Hardy–Weinberg equilibrium [41–43], so *SR* is expected to occur at equal frequencies in males and females. We found no difference in frequencies of *SR* in fathers and mothers (overall *SR* frequency: males, 7.0%; females, 6.7%). As there is no overall deficit of *SR* in fathers, it is unlikely that an inability to detect *SR* fathers caused the observed cline. Taken together, our experiments demonstrate a north–south cline in frequency of polyandry across the USA. Furthermore, combined with previous evidence that female remating frequency in *D. pseudoobscura* is heritable [44], our results show that this cline in polyandry is genetically determined.

Our survey of *SR* frequency in eight populations and our formal analysis of previous data strongly support the conclusion by Dobzhansky [26] and others [27] that a latitudinal cline in *SR* exists across the USA. This cline appears to have remained stable for the past 75 years. The cline in polyandry will be an important driver of this cline. Theoretical work predicts that meiotic drivers could have their transmission inhibited by reduced sperm competitive success, and so variation in rates of polyandry could generate variation in the abundance of meiotic drive [11,45]. For this hypothesis to apply in nature, the driver must significantly reduce the success of male carriers in sperm competition, and sperm competition should occur at a high enough rate to be important. In addition, females should not be able to discriminate against meiotic drive males prior to mating, otherwise mate discrimination against drive-carrying males would be expected to be more important than sperm competition. There is considerable evidence that *SR* in *D. pseudoobscura* fulfils all these conditions. Males carrying *SR* are poor sperm competitors, both in the experiments presented here and as shown in previous studies [28]. This study suggests that *SR* males are particularly poor sperm competitors when competing against males from the northern, more highly polyandrous populations. Females are often polyandrous in nature [32,46], and show no evidence of being able to discriminate between *SR* and *ST* males prior to mating [47,48]. Laboratory experimental evolution studies have shown that in populations where females were allowed to remate, *SR* declined in frequency, whereas in populations where polyandry was prevented, *SR* spread rapidly [4]. As polyandry directly regulates the frequency of *SR* in laboratory populations, the evidence presented here that *SR* is rare or absent in populations with a high level of polyandry, and common in populations where polyandry is more rare, strongly suggests that polyandry also determines the frequency of *SR* in natural populations.

Alternative explanations for the distribution of *SR* have been suggested. Meta-population dynamics is one possibility, with subpopulations carrying high frequencies of drive repeatedly going extinct [49]. However, this would predict that very high frequencies of drive should be observed in local populations, and local extinctions should be common, but at present there is only one tentative report of this [50]. It is also notable that this dynamic would create a checkerboard pattern of presence and absence, rather than the observed clinal variation. [14]. A second suggestion is decreased fitness in females, particularly homozygotes, owing to the accumulation of deleterious mutations within the inversions on the *SR* chromosome [27,43]. Such decreased fitness is seen in the *SR* chromosome of *Drosophila recens* [51], and low fitness of homozygotes is found in *t* haplotypes in mice [52]. However, the evidence for a cost to females of carrying *SR* in *D. pseudoobscura* is poor [14,22,27,53]. Moreover, the one study that found low fitness of female *SR* carriers found that *SR* females were more successful at lower temperatures [53], and so cannot explain why *SR* is rare in the north. Alternatively, *SR* might decrease the fertility of males more at lower temperatures, thereby preventing *SR* persistence in colder areas. However, although male fertility does interact with temperature, *SR* males are *less* fertile at higher temperatures, making this an unlikely explanation of the observed cline in *SR* [22]. Further suggestions, such as increased vulnerability to parasites or lower overwinter survival of *SR*-bearing flies, have been put forward [22,27]. However, at present none are supported by data from either the laboratory or the field.

It is currently not possible to completely eliminate the possibility that the observed correlation of *SR* and polyandry is due to some unknown additional factor that directly influences both, or arose simply by chance. However, there is strong theoretical [11,29] and empirical evidence [4,28] that polyandry reduces the success of *SR* in populations. There is also a lack of experimental support for the major competing theories for the control of *SR* frequency in natural populations [14,22,27], and little evidence that these alternative factors correlate with latitude in a way that could create the observed stable cline in *SR*. Hence, by far the most parsimonious explanation for the clinal distribution of *SR* is that it is maintained by the underlying cline in polyandry reported here, providing a potential solution to a 75-year-old puzzle in population genetics [21].

Polyandry may play a major role in controlling the abundance of many SGEs in nature [13]. Evidence from mice supports this hypothesis [52]. The population dynamics of autosomal meiotic driving *t* alleles in mice have been investigated for decades [54]. Models commonly predict *t* allele frequencies 10 times higher than those found in natural populations (the ‘*t* frequency paradox’) [55]. Recent work using models parametrized with extensive laboratory and field experiments found that the dynamics of *t* alleles in a natural population of house mice could only be explained by the transmission disadvantage owing to sperm competition resulting from polyandry [52]. Clinal distributions may also be common in SGEs. Several other sex chromosome meiotic drivers are distributed along latitudinal clines, being commoner in southern populations than northern ones. These include drivers in *Drosophila persimilis* [27], *D. subobscura* [56] and *D. recens* [51]. The endosymbiont *Wolbachia* also seems to be clinally distributed in the weevil *Curculio sikkimensis* [57]. It is possible that these clines may also be caused by underlying clines in polyandry, although this has not been investigated. Our conclusion that polyandry can determine the distribution of an SGE in nature is supported by recent work in another *Drosophila* species, *D. neotestacea* [50]. In *D. neotestacea*, another X-chromosome meiotic driver shows a latitudinal cline across North America, being rare in Canada [58]. A similar cline in polyandry has been found in this species, with northern females remating more frequently, and degree of polyandry covaries with the frequency of meiotic drive in natural populations. *Drosophila neotestacea* is only distantly related to *D. pseudoobscura*, and the meiotic drivers evolved independently. The discovery of similar patterns in both species is strong evidence that polyandry can protect populations from SGEs in nature. Hence it is likely that geographical differences in degree of polyandry are generally important in determining the frequency of many SGEs in the wild. Meiotic drivers may be particularly likely to be controlled in this way, because damaging sperm is an essential part of the drive mechanism [14].

If the abundance of sex chromosome drivers is partly determined by the level of polyandry, this could have an impact on the whole population. The presence of meiotic drivers can cause females to evolve increased rates of polyandry [44], which can in turn promote the evolution of male counter-adaptations that suppress female remating [59], increasing the level of sexual conflict throughout a population. Most obviously, sex ratio distorters can influence population sex ratio [14]. Population sex ratio is a key ecological parameter, influencing factors such as effective population size [60], population growth rates [61], mate competition and mate choice [62]. High frequencies of *SR* result in female-biased population sex ratios, and the population sex ratio in *D. pseudoobscura* can be directly determined by the frequency of *SR* [63]. Laboratory studies tracking the spread of *SR* through experimental evolution populations also show that the frequency of *SR* can control population sex ratio [4]. Here we argue that, although correlational, our results strongly suggest that the level of polyandry also determines the frequency of *SR* in natural populations, and the population sex ratio in *D. pseudoobscura* is therefore ultimately determined by the frequency of polyandry. This is a remarkably powerful impact of individual female mating decisions on the ecology of populations [64].

Our results demonstrate the existence of a stable cline in polyandry across the USA. This cline is heritable, acts through female mating behaviour and may be maintained by current selection on the frequency of polyandry. Furthermore, these differences in polyandry across populations seem to control the frequency of a sex-ratio-distorting SGE in nature, ultimately determining population sex ratio at a landscape scale. Many other SGEs can control population sex ratio [13,62,65] and impair sperm production [20], making them vulnerable to control through polyandry [11]. Hence, although polyandry can increase conflict between individuals [2,7] and repress the evolution of sociality [9,10], it also has the potential to promote harmony within the genome by suppressing the spread of SGEs.

## Data accessibility

No large datasets are involved in this work. However, isolines and material will be made available on request.
